# Holistic community-based group parenting programs for mothers with maternal mental health issues help address a growing public health need for a diversity of vulnerable mothers, children and families: Findings from an action research study

**DOI:** 10.3389/fgwh.2022.1039527

**Published:** 2023-01-17

**Authors:** Paul Aylward, Anne Sved Williams

**Affiliations:** ^1^ Action Research Partnerships, Adelaide, SA, Australia; ^2^ Torrens University Australia, Public Health, Equity and Human Flourishing, Adelaide, SA, Australia; ^3^ Department of Psychiatry, Women’s and Children’s Health Network, SA, Australia; ^4^ Faculty of Health and Medical Sciences, University of Adelaide, SA, Australia

**Keywords:** maternal mental health, parenting, holistic, depression, borderline personality disorder, well-being, action research, attachment theory

## Abstract

**Background:**

Maternal mental illness is a major growing global concern which can affect parenting with serious negative implications for offspring. Group-based parenting programs for mothers which both enhance the parent-child relationship and address mental health symptoms in a supportive social setting may optimise better outcomes for mothers and children. The Acorn program in South Australia draws on attachment theory to integrate dance play, reflective diary keeping and therapeutic letters in a holistic program for a diversity of vulnerable mothers and children aged 1–36 months. The program seeks to nurture and enhance parental wellbeing and the quality of the parent-child relationship for mothers experiencing identified mental health illnesses that impinge upon their parenting. This study presents the evaluation of the program and its effectiveness.

**Methods:**

Action research approach for continuous monitoring and program improvement engaging Acorn program staff in evaluation data collection and interpretation of pre and post self-completion measures and standardized observations. Additional data was collected through a telephone interview of attending mothers 6–8 months after program completion to address sustainable impacts on parenting and wellbeing.

**Results:**

The program engaged 353 diverse vulnerable mothers with their children. Many had profound overlapping mental health issues including borderline personality disorder (BPD) and depression. The quality of the parent-child interaction, parental confidence, competence and enjoyment were enhanced; mothers' wellbeing, ability to cope and lasting social supports were augmented. This occurred for a number of “most vulnerable” subgroups including single mothers, mothers with BPD, mothers from non-English speaking households and those with lower levels of education or household income. Mothers reported sustained improvements in their wellbeing, parenting, social and family lives, and feeling closer to their child as a result of participating in the program.

**Conclusions:**

Given the high prevalence of maternal mental health issues and substantial potential negative consequences for mothers and offspring, the Acorn parenting program offers an effective means of addressing this pressing public health issue potentially helping large numbers of vulnerable mothers and their children. This has additional gravitas in the shadow of COVID-19 due to expanded numbers of those experiencing greater parental stress, isolation and mental illness.

## 1. Introduction

Maternal mental illness and particularly maternal depression is common (1) and for many enduring. A recent overview of 80 countries estimated a global postpartum depression prevalence of around 17.2%; the highest rate of 39.96% was found in South Africa, with Australia being around 11.22% (2). The majority of people suffering from depression have at least one comorbid mental disorder (3), and an array of other specific mental health illnesses experienced during motherhood including psychosis, bipolar disorders and borderline personality disorder (BPD) contribute to an overall persistently high prevalence of maternal mental illness (4). While postpartum depression can be experienced by women up to twelve months after the birth of their child (5), experiencing depression is not limited to the postpartum period; an Australian study has found maternal depression to be more common when the child passes toddler age than in the first twelve months postpartum (6), and severe postnatal depression elevates mothers' risk of continuing to experiencing depression in the following decade (7).

Mothers with maternal mental illnesses may experience a range of issues including depressed mood and sadness, mood swings, difficulties coping, feelings of guilt, shame and worthlessness, extreme concerns about their baby, anxiety, a loss of interest in daily activities, a sense of overwhelming loneliness and feeling alone in their experiences, and a strong sense of stigma (8–14) Experiencing mental illness also increases the risk of family conflict and relationship breakdown (15) and maternal suicide (16, 17), a leading cause of maternal death in the peripartum period (18) with elevated risk for those experiencing BPD (19–21).

The compromised emotions many mothers with maternal mental illness experience can distort emotional communication with their children. Subsequently mothers' enjoyment of the parental role may be reduced affecting a delay in developing competency in parenting skills and potentially creating a detached, mechanistic caregiving experience (22). Given these difficulties, it is predictable that maternal mental illness is associated with low perceived parental self-efficacy (PPSE) (23, 24), and low maternal confidence (25). PPSE refers to the capabilities parents believe they have to successfully organise and execute situation specific parenting tasks (26), and this is positively associated with actual parenting competence (23). Low maternal confidence experienced in the early postpartum period by mothers with maternal mental illness has also been shown to persist for several years to pre-school age despite improvements in the mother's mental health, and may continue to have a negative effect on the mother-child relationship (25, 27). Postpartum depressive symptoms are also associated with higher parenting stress (28–30), and “Parental Distress” in particular has also been found to predict later depressive symptoms (31). However, despite these myriad issues there is qualitative evidence that women with severe mental illness may value motherhood more highly as a central positive influence in their lives (12).

The profoundly negative effects maternal mental health can have on offspring have been broadly documented (32–40). However, this causal pathway has been shown to be mediated by the quality of parenting behaviours (33, 41–43). There is a large body of research associating a broad range of maternal mental illnesses with negative parenting behaviours, disengaged parenting and poorer mother-infant interaction during infancy, with most of this work addressing maternal depression (41, 44–57). Stress in the parenting system has also been specifically shown to impinge on the quality of parenting behaviour (58), the quality of the developing relationship between mother and infant (35), and the meaning and satisfaction these mothers derive from caregiving (59). The quality of mother-child interaction has been shown to predict the child's later positive cognitive and socio-emotional development, and academic competence in middle childhood (60–64). Moreover, an important predictor of parenting behaviour is how parents were parented themselves (65, 66), highlighting the potential endurance of unchecked problems through generations.

There is accumulated evidence, that a variety of psychosocial and psychological programs including peer/social support, group treatment, and non-directive counselling where mothers are encouraged to explore their feelings and find their own solutions benefit mothers with postpartum depression (67–69). A major challenge here is that treating the mother's mental illness alone does not necessarily help enhance an impaired parent-child relationship and its negative consequences for the child (70–76). Notably, studies with mothers in remission have demonstrated that impairments in parenting developed during acute maternal depression can persist (77, 78). However, building parental confidence and PPSE, can have a positive influence on healthy parenting practices (79), and interventions that successfully enhance the mother-infant relationship and build parental confidence have been shown to provide a protective buffer for the effects of maternal mental illness and subsequent compromised child development (75, 79, 80).

Given the potential to modify parenting behaviour, there is a strong case for developing effective parenting interventions for mothers with maternal mental illness to mediate the effects of their illness on child outcomes (33). Interventions that utilised both maternal-child interaction guidance and psychotherapeutic group support can significantly improve the mother's mental health, parenting and child development outcomes (81). The evidence therefore suggests the need to combine strategies to address mental health symptom relief with strategies to enhance the quality of the parent-child relationship. In the latter case integrating activities that enhance mother-infant interaction, build parental confidence/perceived parental self-efficacy, and encourage sensitive parenting and maternal bonding may be of particular benefit (74, 82, 83). Holistic group-based parenting interventions may offer additional social support along with the potential for a mutual learning environment where skills and strategies can be practiced (84). This composite approach may optimise the likelihood of better outcomes for mothers, children, and families (73, 75, 85, 86). However, it is important to highlight that studies to date have tended to have relatively small samples of often homogeneous groups of women, focussing on a small number of outcomes (81). It is against this background of the need for an effective blended composite group-based approach implemented in the broader diverse community of mothers with maternal ill health that the Acorn program in South Australia and its concurrent evaluation emerged.

The effectiveness of a community-based program delivered to a relatively heterogeneous client base should include consideration of identified vulnerable subpopulations. Mothers who are single have been found to be particularly vulnerable to maternal mental illness (87), including chronic stress and depression (88–90). Migrant mothers to higher income countries are also at increased risk particularly where there are language difficulties (91, 92) with this applying to both clinical and non-clinical levels of postpartum depression (93, 94). In both cases these risk factors may be compounded by greater social isolation and loneliness, both of which are commonly associated with significant mental illness and poorer mental health (95–101). Income and education have been found to have significant negative associations with depression severity among mothers of young children (102), and socioeconomic factors have been found to moderate the relationship between maternal mental illness and negative parenting practices with the association tending to be stronger in lower socio-economic groups and where the mother has lower formal education (41, 54). A systematic review addressing maternal BPD has concluded that these mothers are more likely to engage in maladaptive interactions with their offspring (103) and qualitative research has identified the emotional intensity, social isolation and lack of positive parenting models for these mothers warrant parent-focussed support (104). Finally, postpartum depression has been found to be more prevalent in multiparous mothers of young children than their nulliparous counterparts, with this being attributed to the greater psychological demands of parenting more than one young child (102, 105, 106).

The evaluation of the Acorn program has therefore examined its effects for a diverse client base of mothers with mental illness including the specific subpopulations: single mothers, those who speak a language other than English at home, those whose main source of household income is Government benefit, and mothers whose education level has not progressed beyond Year 12 school level. Mothers who indicate BPD, and those with more than one young child of the ages targeted by the Acorn intervention were also considered as additional vulnerable subpopulations to consider in order to assess the value of the program.

## 2. Program overview

The Acorn parenting program is a face-to-face group-based holistic intervention of 15 weekly sessions of two hours duration provided in South Australian community venues (mostly child care centres) to help mothers of young children (0–3 years) who are experiencing chronic, and in many cases profound mental health challenges which impinge upon the quality of their parenting. Mother-child dyads are referred by a broad range of sources including Child and Family Health Services, Perinatal and Infant Mental Health Services, general hospitals, and private psychiatric and psychology consultants. This enables a diverse range of mothers to be directed to the program by their referrers with the only exclusion criteria being a current diagnosis of a psychotic disorder which might render group work with other mothers inappropriate. Acorn groups were conducted across fifteen geographically dispersed locations in metropolitan and outer metropolitan Adelaide suburbs with the program being delivered in “waves” twice a year to between 4 and 10 groups at a time.

The theoretical underpinnings of the Acorn program were informed by attachment research which reinforces how the quality of the parent child attachment has a significant role in the life trajectory of the child (107) and highlights the importance of addressing the origins of disorganization (108), encouraging parental sensitivity (109) and developing reflective functioning (110). This therapy allows the women to experience a relationship that is safe, curious and reflective, while assisting the processing and integration of her past parenting and mental health history (111).

Each Acorn session incorporates three evidence based “primary components”. “Dance Play”, “Reflective Journaling” and “Therapeutic Letters”. Dance Play activities are designed to encourage meaningful mutually enjoyable playful interactions between mothers and infants (112–115) and to nurture the child's agency to visibly emerge while developing the mothers’ abilities to recognize and respond to their child's cues, needs and behaviours as they build new repertoires of play and engagement (116). Dance play activities and songs engage all attending children including a “Hello” song at the beginning of the session in which each participating child's name is sung by the group, but Dance Workers also draw from compiled program activities and songs tailored to the developmental ages of the attending children drawing on Clark and Dawson's conceptual stages of “being, thinking and doing” (117). “Reflective Journaling” (118–120) seeks to encourage mothers' strength based reflection on their perspectives and lived experiences surrounding their parenting and mental health, including exploration of core relational conflicts (121). This component also provides a forum where mothers can share and explore their experiences with their peers, helping them to understand that they are not alone in their struggles and encouraging supportive friendships to develop. “Therapeutic letters” (122, 123) sent as e-mail correspondence between sessions act as a mode of extending reflections from the therapist to patient beyond formal sessions. Parents are supported in a “non-judgmental” safe environment to overcome blocks in their ability to read, interpret and respond to their infant's cues accurately and appropriately.

The groups are staffed by a dance and movement specialist (qualified dance practitioners with formal post graduate qualifications in this area) who facilitates Dance Play, a Mental Health Clinician (a psychologist, social worker, or mental health nurse, trained in maternal and infant mental health and attachment theory), who facilitates the compilation of Therapeutic Letters, and a Family Support Practitioner (qualified social worker) who leads the journaling and reflection components of the groups.

Following the Acorn group session, the staff team conducts a ninety-minute group supervision and debriefing session facilitated by the Mental Health Clinician in which reflections on each dyad are shared to inform the tailored Therapeutic Letter, and any staff concerns are discussed. The Mental Health Clinicians also provide individual supervisory support for the other team members, and are supported themselves by an external clinical supervisor. An Acorn Coordinator, a qualified social worker with knowledge of attachment theory, organizes group referral allocation and program promotion activities, with the whole program being overseen by a Program Manager.

Peer meetings of Acorn Dance Workers, Family Support Practitioners and Mental Health Clinicians are regularly conducted to enable shared reflections on practice and maintain program fidelity within their own discipline. Whole of program team meetings are conducted at the end of each program year in which evaluation findings are also presented and discussed.

Facilitator capacity is enhanced through internal training using developed program resources and induction including observations of existing Acorn groups followed by staff debriefing. Externally sourced training needs are reviewed periodically through a Critical Reference Group (124) comprised of program stakeholders and representatives of referring agencies, and this has included formal training in therapeutic letter writing, perinatal infant mental health, the Circle of Security (125), attachment theory, Marte Meo training (126), understanding personality disorders, strengths based practice, and developing cultural competence.

**Figure 1:**
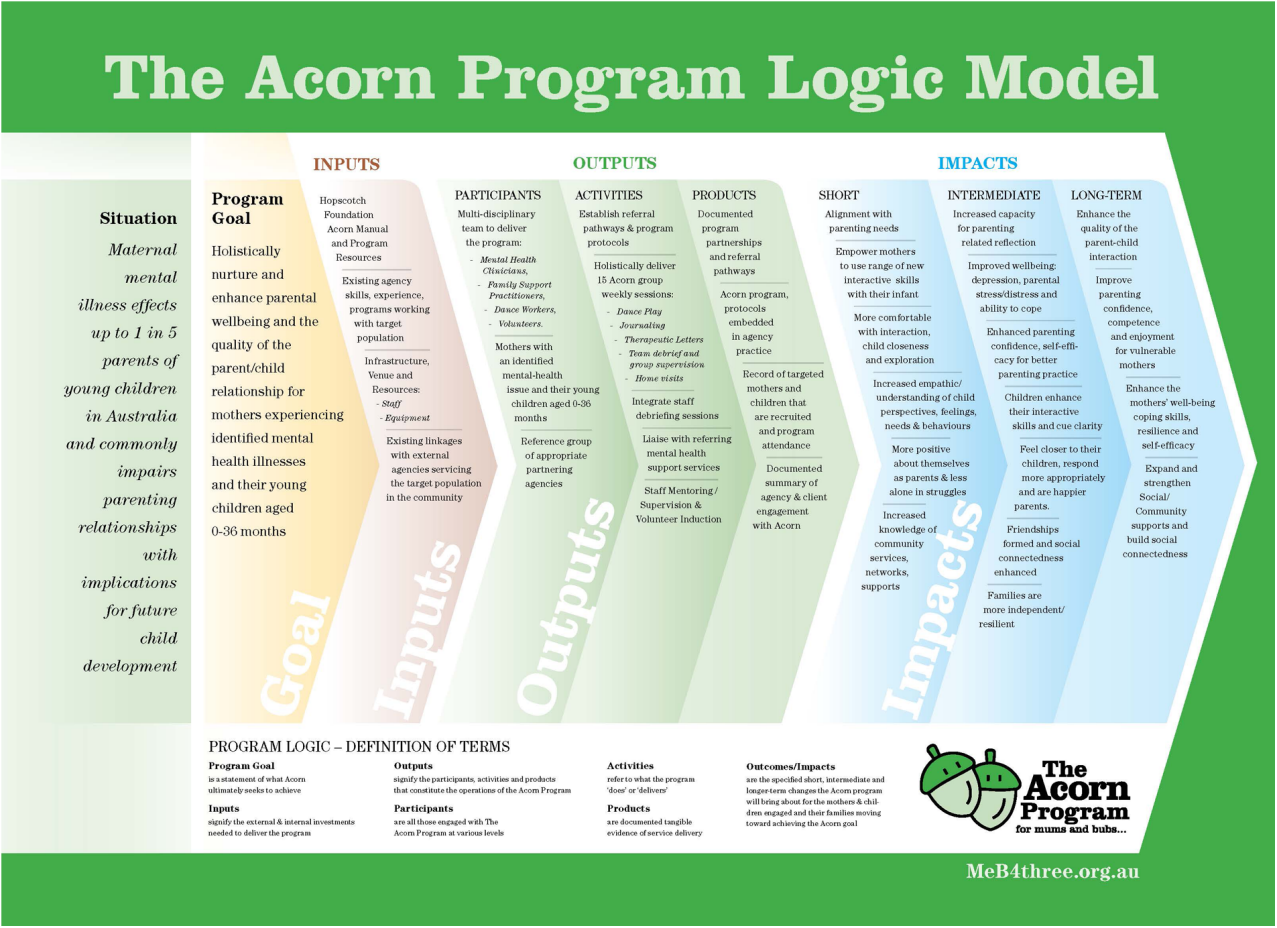
“*The Acorn Program Logic Model”: source:* Chance S. *The Acorn Program: from small things big things grow*. 2021. South Australia: The Hopscotch Foundation PTY. https://meb4three.org.au/acorn/.

The Acorn program inclusively workshopped and designed a program logic stipulating a range of short, intermediate, and longer-term desired Acorn outcomes (Figure 1). The stated goal of the Acorn program is: “*To holistically nurture and enhance parental wellbeing and the quality of the parent-child relationship for mothers experiencing identified mental health illnesses and their young children aged 0*–*36 months*”*.* The four longer-term collectively agreed objectives of the program are: *to enhance the quality of the parent-child interaction; to improve parenting confidence, competence and enjoyment for vulnerable mothers; to enhance mothers' wellbeing, coping skills, resilience and self-efficacy; to expand and strengthen Social/Community supports and build Social Connectedness.* The program logic and full program details are included as part of the Acorn Manual available through the MeB4three website (116).

## 3. Design, materials and methods

This paper presents the summative quantitative process and outcome findings (pre-post measures and follow-up interviews) from the first eleven Waves of Acorn over a period of 5.5 years. Qualitative findings from the open-ended questions and focus groups exploring in-depth experiences of mothers on the program will be reported in a separate paper.

The evaluation adapted an action research approach (127, 128), providing evaluation training to Acorn service providers who administered the battery of evaluation tools pre and post each program wave to attending mothers. There were no incentive payments for participating in the evaluation and mothers were informed that while their participation may not benefit them directly, their answers may inform future program refinement. All mothers who had enrolled were eligible for the pre-measures and all mothers who had not formally withdrawn from the program were eligible for post-measures regardless of how many sessions they had attended. Client self-completion tools were answered individually and sealed in envelops to ensure and demonstrate confidentiality before being sent to the evaluator for data entry and analysis. The findings were analyzed between waves by the evaluator and presented to the Critical Reference Group. Evaluation was a standing agenda item here, informing a collective and democratic process of interpretation, reflection, and planned revisions to program delivery to be formulated progressively in order to refine and improve program implementation and evaluation in subsequent waves. Representatives from each of the discipline groups facilitating Acorn also provided their group reflections for discussion during these meetings to further inform improvement to future practice in a continuing cyclical learning process (127, 129).

Given the vulnerability of Acorn participants, and the concern to retain client engagement in Acorn, ensuring that the compounding burden of evaluation demands was acceptable and manageable was a primary concern, with this being monitored during client focus groups and regular feedback from Acorn staff interviews. The standardised psychometric tools were selected based on: the ability of the tool to provide reliable, valid, and relevant indications of objectives being met; the appropriateness of the tool for application with vulnerable Australian mothers of children aged between 0 and 36 months of mixed educational and cultural backgrounds; the ease of administration and completion for participating subjects in a community-based group setting. The Bellberry Human Research Ethics Committee scientifically and ethically reviewed all aspects of the evaluation.

The tools applied throughout the 11 waves of the program were:

*The Parenting Stress Index Short Form [PSI-SF]* (130) is a 36 item scale measuring “Total Parenting Stress” with the following 12 item subscales: “Parental Distress”, the distress parents experience in their role as a function of personal factors that are directly related to parenting; “Parent–Child Dysfunctional Interaction”, the degree to which the mother perceives their child as not meeting expectations, with scores in the 96th percentile suggesting potential for abuse; “Difficult Child”, the parent's perception of the behavioural characteristics of the infant that makes him/her either easy or difficult to manage. Scores are interpreted relative to percentiles from a normative sample for each subscale and the total provides indications of “Clinically Significant”, “High”, “Normal Range” or “Low” levels for each respondent. The scale has been translated into over 40 different languages and validated across a broad range of cultures and populations (ibid).

The *Patient Health Questionnaire [PHQ-9]* (131) provides a criteria-based indication of depressive disorders and measure of depression severity. The PHQ-9 is the nine-item depression module taken from the larger Patient Health Questionnaire (PHQ) which is a self-administered version of the Primary Care Evaluation of Mental Disorders (PRIME-MD) diagnostic instrument for common mental disorders. The PHQ-9 focusses exclusively on the 9 diagnostic criteria for DSM-IV depressive disorders, scoring each as “0” (not at all) to “3” (nearly every day). A total score of ≥10 has a demonstrated sensitivity of 88% and specificity of 88% for major depression, with scores of 5, 10, 15 and 20 validly demarcating the lower limits indicating “minimal symptoms”, “minor depression/major depression mild”, “major depression moderately severe”, and “major depression severe”. The tool includes a supplemental scale addressing the level of functional difficulty respondents may have in their work, home or social life as a result of any indicated symptoms with this ranging from “not difficult at all” to “extremely difficult”.

The *Karitane Parenting Confidence Scale [KPCS]* (132) is used to assess the unidimensional construct Perceived Parental Self-Efficacy (PPSE) in parents with infants aged 0–12 months. To ensure eligibility for this measure pre and post scores were analysed for children aged 8 months or less on commencing the program. This 15-item scale was developed using an Australian population of mothers including participants from culturally and educationally diverse backgrounds. Grounded in Bandura' self-efficacy theory (133), the items in the KPCS follow his recommendation to be of a “task specific” nature, which is considered to imbue a greater sensitivity and predictive validity for scales addressing “self-constructs” (134, 135). Higher scores indicate greater Perceived Parental Self-Efficacy with clinical cut-off scores: “severe clinical” range <31, “moderate clinical” range 31–35, “mild clinical” range 36–39, and “non-clinical” range ≥40. Cronbach's alpha for the KPCS has been calculated as .81 and test-retest reliability .88, with sensitivity and positive predictive power calculated at .86 and .88 respectively (134).

*Client Self-Completion Questionnaires*, semi-structured pre and post tools including demographics, process and impact Likert scales and open questions inviting further comments on the “closed” item responses. The pre-measure included Likert Scale items for mothers to indicate personal reasons for attending Acorn (e.g., *I would like to be able to interact better with my child/children).* The post-measure included a Likert scale operationalising indicators addressing each objective (e.g., *I interact better with my child because of attending this group*; *The group has helped me to cope better as a parent; I made friends with other parents in the group*); and a separate scale under the heading “*For each area, please indicate the extent to which your current parenting abilities or experiences have improved (if at all) as a result of attending the group*”, with this including items addressing parental understandings, feelings and reflections; all items appear in Table 5. Scales included negatively worded items (e.g., *The group was not very helpful for me*), to provide an indication of diligence in client response. Mothers were also asked to indicate the extent to which they would recommend the Acorn program to their peers. The questionnaires were piloted with a small group of mothers prior to the evaluation.

A six-eight month “*Follow-Up*” *Telephone Interview* was applied with mothers from Waves 1–9 who had consented to be contacted for the purpose at the beginning of the program. This was conducted by a trained female independent interviewer who had professional experience in social work and maternal mental health. The interview schedule included Likert scale items from the post *Client Self-Completion Questionnaire* which were read verbatim to participants to enable direct comparisons with responses provided on completion of the program. The interviews lasted between 25 and 60 min.

Two additional tools were introduced as the program developed:

The *McLean Screening Instrument for Borderline Personality Disorder [MSI-BPD]* (136), applied in wave 4, is a 10 item self-report measure addressing BPD based on *DSM-IV* BPD criteria. Each endorsed item scores 1 point with a score of 7 or more indicating likely BPD. The tool has demonstrated good sensitivity (.81) and specificity (.85). For young adults between the ages of 18–25 years diagnostic efficiency of the tool was higher; sensitivity .90 and specificity .93. The tool has good internal consistency with a Cronbach's alfa of .74. The MSI-BPD was applied as a pre-measure only to provide indications of borderline personality disorder (BPD).

The *Nursing Child Assessment Satellite Training* [NCAST] *Parent-Child Interaction Teaching Scale [NCAT**]*** (137), introduced in Wave 6 is a 73 item observational tool used to measure the quality of the parent-child interaction with children up to 36 months. This is comprised of a number of subscales with the most reliable measures over time being the composite scales of “Caregiver-Infant Total” (the overall quality of the interaction), “Caregiver Total” (assessing the quality of the mother's contribution to the interaction, including a subscale of items addressing the mother's “sensitivity to cues”), and “Child Total” (assessing the child's contribution, including a subscale of items addressing “clarity of cues”). The NCAT is theoretically grounded in the Barnard model (138, 139) conceptualising the caregiver-child interaction as a reciprocal dialogue in which quality is defined and created by the contributions of both participants (140). The NCAT has acceptable internal consistency for the composite scales with Cronbach's alphas of .87 for “Caregiver Total”, .81 for “Child Total”, and .88 for “Caregiver/Infant Total” (141). A normative database has been established for the NCAT using a large US sample of 1,887 dyads from a range of ethnic, educational, and familial backgrounds and this has been found to be appropriate for other culturally diverse Western populations (137). Applied from Wave 6–10 the NCAT was administered in mother's homes, using video of dyad interactions addressing child age-specific tasks. Pre and post assessments were balanced using different annually certified Mental Health Clinicians all of whom were accredited through a three-day structured training program to apply the tool for clinical and research purposes having achieved inter-observer reliability score of >90% (142).

## 4. Analyses

Data were analysed using IBM SPSS Statistics Version 25. Paired samples *t* tests were employed to assess change from pre to post measures for the standardized tools with a two-tailed statistic used as more conservative test, and effect sizes are presented using Cohen's *d* (143). More vulnerable subgroups of mothers identified from the literature were considered individually, and where samples were relatively small (20 cases or less), a robust bootstrapping technique was employed and the bias corrected and accelerated 95% confidence interval (BCa 95% CI) estimated (144, 145). Where available, scores are compared with normative data for interpretive purposes. A “reliable change index score” (146) was applied for the KPCS to determine clinically meaningful changes in PPSE. A clinically significant improvement in depression from a clinical to non-clinical score for the PHQ-9 was also calculated by obtaining a post-treatment score of ≤9 combined with an improvement of 50% or more (131).

While some mothers went on to repeat the program (including twelve who had originally withdrawn from it), the findings presented are for the first attendance of the Acorn program to provide clear indications of its worth as a single intervention for the targeted population**.** Where completing mothers had two children attending Acorn, unless otherwise stated the figures refer to their youngest child for clarity.

## 5. Results

### 5.1. Client participation & profile

The total number of enrolled mothers (*N*^e^) to the program was 493 (Mean age 30.8 years, SD 5.6) with 520 children (mean age 9.1 months, SD 7.1). The number of individual mothers completing the program (*N*) was 388 (78.7%), with 353 (71.6%) providing both pre and post measures of the client self-completion questionnaire (*n*). Of the 27 mothers who enrolled with two children, 19 completed the program. Mothers who completed the program are referred to as “Acorn mothers” in this paper. 105 (21.3%) mothers withdrew from the program. Reasons obtained for withdrawal from Acorn were largely innocuous and unrelated to the program although twenty-six mothers indicated a health reason for withdrawing. There does not appear to be a systematic reason for client withdrawal: the demographic and psychological profile of “withdrawing” mothers (depression, PPSE, Parenting Stress, BPD) was very similar to that of retained Acorn mothers, and the proportion of withdrawing dyads performing poorly in the pre-measure of the NCAT mirrored that of retained dyads. Withdrawal was not related to the geographical location of the group attended. Client personal objectives for attending Acorn were also very similar for withdrawing and retained mothers. Client withdrawal has declined over the duration of the program from 42.5% in Waves 1–4, to 23.0% in Waves 8–11.

The follow-up telephone survey of completing mothers yielded a response rate of 37.8% (135 mothers) - (62.8% of consenting mothers who could be contacted). The profile of interviewed mothers was very similar to that of the broader group of mothers although they were slightly older (M 31.7 years, SD 5.38) and more likely to have more than one child (44.0% vs. 34.8%, respectively).

Acorn recruits a wide variety of participants from a broad spread of socio-economic, cultural, familial, and educational backgrounds, and has enrolled a profile of mothers where depression, poor perceived parental self-efficacy (PPSE), and high parental stress are overlapping causes of concern and which for many indicate the need for clinical intervention (Table 1). Nearly nine in ten mothers had symptoms of depression and 30.2% indicated moderate-severe major depression, of whom 84.5% (123 mothers) also indicated “clinically significant” or “high” levels of “Parental Distress”. One hundred and thirteen (30.4%) mothers indicated BPD on commencing the program from Wave 4, with 81 (71.6%) of these completing the program. Just under half of the 113 mothers who indicated BPD (49.6%) also indicated moderate-severe major depression. Acorn dyads scored relatively low in the NCAT observational scale addressing the quality of the parent-child interaction where 36.6% scored >−1 SD from the normative mean in their “Caregiver-Infant Total”.

**Table 1:** Baseline characteristics of acorn participant enrolments.

Demographics (*N*^e ^= 493 mothers; 520 children)		Baseline Psychological Profile Indications	
Characteristic	*M* (*SD*)	Characteristic	%
Mother's age (years)	30.8 *(5.6)*	Depression: PHQ-9 [*n* = 486]	
Attending Child's age (months))	9.1 *(7.1)*	- *Depression Symptoms*	87.2
		- *Minimal Symptoms*	31.3
		- *Minor Depression – Major Depression mild*	25.7
		- *Major Depression Moderately severe*	17.7
		- *Major Depression severe*	12.5
	**%**		
Mother's Education level reached		Perceived Parental Self-Efficacy: KPCS [*n* = 394] (for child ≤ 1 year at pre-measure)	
Yre 10 or lower	8.9		
Yre 11	5.9		
		- *“At least to a Clinical Level”*	80.2
Yre 12	12.4	- *“Severe Clinical level”*	24.4
Tafe or College	39.6		
University	31.1		
Marital Status		Parenting Stress: PSI-SF [*n* = 485] *“Clinically Significant” or “High”*	
Single	15.0		
Separated/Divorced	5.9		
Married/De Facto	76.4	- Total Parenting Stress	16.9
Born Outside Australia	11.9	- Parental Distress	54.4
Non-English Language Spoken at Home	15.1	- Parent-Child Dysfunctional Interaction	12.4
ATSI	2.8	- Difficult Child	12.2
Mother's Employment			
Full-time parent	30.6		
Unemployed/Not working	15.9	Borderline Personality Disorder Indicated MSI-BPD [*n* = 372]	30.4
Currently working	16.2		
On-leave from work	27.0		
Studying	3.4		
Main Household Income Source		Quality of Parent/Child Interaction NCAT > −1 SD [*n* = 191 dyads observed]	
Wages (you or partner)	64.0		
Government Benefit	35.6	*Mother (Caregiver) Total*	37.7
Number of Children		*Child Total*	27.7
One	65.1	*Caregiver/Infant Total*	36.6
Two	26.7		
Three +	8.1		
Mothers with 2+ children ≤36 months	14.6		
Number of eligible Children Attending (*N* = 520)			
Mothers with 1 attending child	94.5		
Mothers with 2+ attending children	5.5		
Attending Child female	47.7		
Attending Child ≤8 months	63.9		

There is a clear alignment of intended program outcomes with the needs of its referred clients; most mothers endorsed a range of Likert statements relating to the Acorn program objectives as their own “personal objectives” for attending the program (Table 2).

**Table 2:** Client personal objectives for enrolling in acorn (*N*^e ^= 493).

	% Agreed (“Strongly”)
I would like to cope better as a parent	93.0 (67.1)
I want to feel better about myself as a parent	90.1 (62.2)
I would like to learn more about parenting	88.9 (46.8)
I would like to get to know other parents	87.3 (47.6)
I need more confidence as a parent	84.8 (54.4)
I would like to be able to interact better with my child/children	83.0 (48.0)
I would like to feel closer to my child/children	78.0 (46.6)
I would like to know more about services and community supports for myself or my family	77.2 (36.1)
I would like our family to have better relationships	75.9 (43.6)
I would like more confidence to access services and community supports if I need them	72.2 (34.0)
I would like to feel more connected to my community	68.9 (29.4)
I would like our family to be more independent and resilient	65.3 (34.3)

### 5.2. Process evaluation

The median number of Acorn sessions attended was 11 (mode of 12). Attending families have engaged well with the program (Table 3). Over nine in ten Acorn mothers agreed that they felt relaxed and safe in the program and that the approach used was respectful and appropriate for them, with clear explanations provided by understanding facilitators using clear comprehendible resources at a suitable pace and venue. This included mothers indicating BPD where 96.3% (78 mothers) agreed they felt relaxed and safe (63.0% “strongly”).

**Table 3:** Process items endorsed by participating acorn mothers.

	% Agreed (“Strongly”)			
	Waves 1–4 (*n* = 113)	Waves 5–8 (*n* = 141)	Waves 9–11 (*n* = 99)	Whole program (*n* = 353)
I was treated respectfully by the Group workers	99.1 (74.3)	99.3 (79.3)	100 (85.9)	99.4 (79.5)
I felt relaxed and safe at the centre	97.3 (57.5)	98.6 (65.7)	100 (72.2)	98.6 (65.1)
What was happening in the Group was explained to me clearly	98.2 (59.3)	98.6 (68.6)	99.0 (64.6)	98.6 (64.5)
The materials/resources I received were clear and understandable	97.3 (51.3)	98.6 (64.5)	100 (65.7)	98.6 (60.6)
This is a good venue for the groups to be held	94.7 (63.7)	98.6 (75.2)	99.0 (68.7)	97.5 (69.7)
The group sessions were delivered at the right pace for me	92.0 (50.9)	94.3 (61.7)	98.0 (60.6)	94.6 (58.0)
The group facilitators understood my issues	91.2 (52.2)	96.4 (64.3)	93.9 (64.6)	94.0 (60.5)
The approach used in the group was appropriate for people like me	86.7 (36.3)	93.6 (49.6)	94.9 (55.6)	91.8 (47.0)
There were not enough opportunities to discuss my experiences of being a parent	27.7 (10.6)	24.1 (5.0)	21.2 (8.1)	23.8 (7.6)
There were too many parents in the group for things to work properly	3.5 (1.8)	2.8 (1.4)	2.0 (1.0)	2.8 (1.4)
The group was not very helpful for me	0 (0)	1.4 (0)	2.0 (0)	1.1 (0)

While positive endorsement of the program was consistently high throughout the program, this improved as the program matured from Wave 5 and this was particularly notable in the increased proportions of mothers “strongly” agreeing with process items between the earlier Waves 1–4 and the later waves 9–11 in Table 3, with *The approach used in the group was appropriate for people like me* improving 19.3 percentage points between Waves 1–4 and Waves 9–11. Eighty-four mothers (23.8%) indicated they would have liked more opportunities to discuss their parenting experiences with this declining as the program matured and refinements where gradually implemented in the reflective journaling component; by Wave 11 three mothers agreed with this item (6.7%) and only one “strongly”.

Over eight in ten completing mothers considered all three principal components of Acorn to be either a “huge help” (for just over half of the attending mothers) or “quite a lot of help”, with 88.6% (312 mothers) indicating this for Dance Play, 86.4% for the Therapeutic Letters and 82.1% for the Reflective Journaling. Talking with other mothers was also considered “a huge help” or “quite a lot of help” by 82.7%. Despite the high rates of approval for the Acorn strategies throughout the program, the perceived helpfulness of strategies has improved as the program has matured and its delivery refined. Comparing Waves 1–4 with Waves 9–11, those indicating that Dance Play was “a huge help” increased from 48.2% to 58.6% and for the Therapeutic Letters from 49.6% to 61.6%. Those asserting that talking with other mothers was a “Huge help” improved from 38.1% to 56.6%. The perceived helpfulness of Reflective Journaling remained consistent throughout the program (54.0% and 54.5% respectively indicating this had been a “huge help”).

All but a single participating mother in the Acorn program indicated that they would recommend the program for other parents like them, with 265 (77.3%) indicating “Yes, with no changes”.

### 5.3. Outcome evaluation

#### 5.3.1. Objective 1: to enhance the quality of the parent-child interaction

Significant improvement was evident across the NCAT dimensions addressing the quality of the mother-child interaction for the 145 dyads observed pre and post Acorn: “Caregiver-Infant Total” with a small-medium effect size (*M*_diff _= 2.67, *t*(144) = 4.39, *p* < 0.000, *d* = 0.37); “Caregiver Total” (*M*_diff _= 1.46, *t*(144) = 3.04, *p* < .01, *d* .29), and “Child Total” (*M*_diff _= 0.95, *t*(144) = 2.63, *p* = 0.01, *d* .25). Significant improvements in the “Caregiver-Infant Total” were also evident across the majority of “most” vulnerable subgroups considered including those where the mother indicated BPD on the MSI-BPD, (Table 4).

**Table 4:** Quality of parent–child interaction outcomes for acorn mothers and vulnerable subgroups (dyads)** waves 6–10.

Caregiver-Infant Total Scores (NCAT)	*N*	*n*	Pre		Post		*t* (*df*)	*p*	*d*	*BCa 95% CI*
			*M*	*SD*	*M*	*SD*				
All	182	145	51.98	7.12	54.65	8.02	4.39 (144)	<.001	.37	—
Non-English spoken at Home*	28	19	50.79	8.25	52.74	5.58	1.32 (18)	.202	.23	*−5.35, .77*
2+ children ≤36 months	28	28	52.50	6.71	56.04	6.69	2.63 (27)	.014	.53	—
Single*	28	17	50.47	8.27	54.47	7.61	2.26 (16)	.038	.48	*−7.85, −.10*
Main household income Government Benefit	61	39	51.92	8.87	54.87	7.20	2.12 (38)	.041	.33	—
School Level ≤ Year 12	39	29	52.21	7.96	54.65	6.63	2.04 (28)	.051	.31	—
BPD Indicated	57	40	52.05	7.02	54.32	6.09	2.33 (39)	.025	.32	—

* Bootstrapping applied.

** Includes 4 dyads of mothers with a second older attending child.

For mothers there were also significant improvements with small effect sizes in: “sensitivity to cues” (*M*_diff _= 0.34, *t*(144) = 2.24, *p* < .03, *d* .24), and for children there was also significant improvement in “clarity of cues” which approached a medium effect size (*M*_diff _= 0.67, *t*(144) = 4.45, *p* < 0.00, *d* .41).

These improvements were reflected in reductions in scores >−1SD below the normative mean. Taking those dyads for whom pre and post measures were observed: for the “Caregiver-Infant Total”, 34.4% scored >−1SD below the NCAST population mean in the pre-measure and 24.8% in the post measure; “Caregiver Total” reduced from 35.2% to 31.1%; and “Child Total” from 27.6% to 17.2% respectively.

Improvements in the Child Total NCAT scores were reflected in the almost universal agreement (98.9%) that *my child has benefitted from my attending the group*, with nearly seven in ten mothers “strongly” agreeing with this item (Table 5). The strength of this response has improved over the duration of the program; those indicating “strongly agree” increased steadily from 65.4% in Waves 1–4 to 72.7% in Waves 9–11.

**Table 5:** Scaled responses: Acorn mothers self completion questionnaire post-measures (*n* = 353) & six-eight month follow-up interview (*n* = 135).

	% Agreed (Strongly)_a_ or % improved (Great Deal/Quite a Lot)_b_	
	Post Measure	6–8 Month Follow-Up
**Obj 1: To enhance the quality of the parent-child 1nteraction**		
My child has benefitted from my attending the group	98.9 (69.1)_a_	95.5 (56.0)_a_
The group has helped me to feel closer to my child	92.9 (51.0)_a_	91.8 (42.5)_a_
I interact better with my child because of attending this group	88.4 (43.1)_a_	91.8 (37.3)_a_
Understanding my child's need to be in a close relationship with me	98.0 (70.5)_b_	91.7 (69.2)_b_
Ability to appreciate my child's perspective of the world	97.2 (77.5)_b_	96.2 (66.2)_b_
Understanding my child's feelings and how they impact on my child's behaviours	96.0 (64.6)_b_	94.0 (61.7)_b_
Ability to respond appropriately to my child's needs and behaviours	95.5 (54.7)_b_	95.5 (50.4)_b_
Identifying my child's needs	94.1 (51.6)_b_	88.7 (53.4)_b_
Feeling comfortable with my child being physically close to me	91.0 (69.7)_b_	77.6 (55.2)_b_
**Obj 2: To improve parenting confidence, competence, and enjoyment for vulnerable mothers**		
I have gained more confidence as a parent by attending the group	87.5 (33.7)_a_	85.2 (35.6)_a_
I learned a lot about parenting from attending Acorn	75.9 (23.9)_a_	76.3 (25.9)_a_
Using range of ways to interact with my child (e.g., through songs, games, activities to do together)	99.2 (79.6)_b_	97.7 (77.4)_b_
Feeling comfortable with my child exploring the world through play	98.3 (77.3)_b_	97.0 (75.9)_b_
Enjoying my role as a parent	97.5 (70.5)_b_	93.2 (63.9)_b_
Feeling comfortable playing and interacting with my child	97.4 (73.9)_b_	94.7 (72.9)_b_
Ability to reflect on how my current health impacts on my relationship with my child	96.6 (67.7)_b_	96.2 (69.2)_b_
Ability to reflect on how my past experiences impact on my parenting	95.7 (68.9)_b_	92.4 (62.1)_b_
**Obj 3: To enhance mothers’ wellbeing, coping skills, resilience, and self-efficacy**		
The group has made me feel better about myself as a parent	90.4 (38.8)_a_	93.3 (43.7)_a_
The group has helped me to cope better as a parent	89.2 (33.8)_a_	89.6 (37.8)_a_
The group has made me more confident to access other family services if I need them	66.0 (17.3)_a_	76.3 (22.2)_a_
The group has helped our family to be more independent and resilient	59.1 (12.8)_a_	67.4 (13.3)_a_
Recognising that I am not alone in the struggles I can face as a parent	99.4 (83.0)_b_	95.5 (77.3)_b_
**Obj 4: To Expand and Strengthen Social/Community Supports and builds Social Connectedness**		
I will (have) share(ed) what I have learnt from the Group program with other parents like me	89.5 (37.6)_a_	89.6 (44.4)_a_
I made friends with other parents in the group	83.8 (38.1)_a_	76.3 (39.3)_a_
I feel more connected to my community because of attending the group	63.9 (13.9)_a_	56.3 (11.1)_a_
I am more aware of other services and community supports for myself or my family as a result of attending the group	62.9 (15.0)_a_	67.4 (17.0)_a_
The group has helped our family to have better relationships	62.3 (18.1)_a_	76.9 (24.6)_a_

Over nine in ten completing mothers indicated that because of participating in Acorn they had improved across all items related to the quality of the parent-child interaction addressed in the Client Self-Completion Questionnaire (Table 5).

A culmination of improvements in these parenting areas may have resulted in 312 mothers (88.4%) agreeing (including 152 “strongly”) that *I interact better with my child because of attending this group.* The proportions of mothers strongly agreeing with this item improved as the program matured, from 37.2% in Waves 1–4 to 50.3% in Waves 9–11. Moreover, most mothers (328, 92.9%) indicated that *the group has helped me to feel closer to my child* (with 180 mothers strongly agreeing). Just over nine in ten mothers also agreed that they felt more comfortable with their child being physically close to them, with just under seven in ten indicting this had improved “a great deal” or “quite a lot.” The above improvements were also consistently reported by mothers six-eight months after completing the program providing indications of sustained program impact in these areas.

These findings were also similar in relation to those 19 completing mothers who brought a second older child to Acorn, with 78.9% agreeing that *I interact better with my child because of attending this group* (7 “strongly”), and 84.2% indicating that *the group has helped me to feel closer to my child* (8 “strongly”).

#### 5.3.2. Objective 2: to improve parenting confidence, competence, and enjoyment for vulnerable mothers

The desire to improve parenting confidence was specifically indicated by 84.8% of attending mothers with over half feeling this strongly prior to attending the program. On completing Acorn, 309 mothers (87.5%) indicated that *I have gained more confidence as a parent by attending the group* (one third “strongly” agreeing) with this applying equally to parents of older and younger eligible children completing Acorn. Strength of agreement improved as the program matured, from 23.9% “strongly” agreeing in Waves 1–4, to 43.4% in Waves 9–11. Reported improvements in parental confidence was sustained six-eight months after completing the program with a slight shift toward strongly agreeing that this was the case.

This improvement was mirrored in findings from the KPCS for those 221 mothers with children who were aged one year or less on completing the program. Perceived parental self-efficacy (PPSE) has significantly and substantially increased for mothers attending Acorn with a medium effect size (*M*_diff _= 3.24, *t*(220) = 8.55, *p* < .0001, *d* .56). Taking those for whom pre and post measures were available, the proportion of mothers in the “non-clinical” range more than doubled in the post-measure (from 17.6% to 35.7%) and the proportions of mothers in “severe clinical range” more than halved (from 24.9% to 9.5%). Sixty (27.1%) mothers improved their scores by six or more points on the scale in the post measure signifying a “clinically reliable improvement” (146) in PPSE. Significant improvements in PPSE were found for the diversity of more vulnerable Acorn mothers (Table 6) and particularly for those who also had additional children aged 3 years or less, where the effect size for their youngest child was large (*d* .75). Significant improvements in PPSE occurred consistently with medium effect sizes at different time groupings across the 5.5 years duration of the program (Table 7).

**Table 6:** Perceived parental self-efficacy (PPSE) outcomes for acorn mothers and vulnerable subgroups.

KPCS (child ≤8 months on commencement)	*N*	*n*	Pre		Post		*t* (*df*)	*p*	*d*
			*M*	*SD*	*M*	*SD*			
All	248	221	33.95	5.78	37.19	4.66	−8.55 (220)	<.001	.56
Non-English spoken at Home	32	30	33.03	7.43	37.37	4.86	−3.03 (29)	.005	.58
2+ children ≤36 months	43	35	32.00	6.53	36.91	4.54	−4.55 (34)	<.001	.75
Single	35	31	35.26	4.48	37.45	4.21	−3.33 (30)	.002	.49
Main household income Government Benefit	72	61	34.81	4.76	37.09	4.49	−4.48 (60)	<.001	.48
School Level ≤ Year 12	60	49	34.59	5.29	37.20	4.24	−3.59 (48)	.001	.49
BPD Indicated	46	41	33.22	5.58	36.24	5.03	−3.70 (40)	.001	.54

**Table 7:** Standardized self-completion tools outcomes by clustered waves.

Waves	KPCS					PHQ-9					PSI-SF (Total)				
	*n*	*M*_diff_	*t* (*df*)	*p*	*d*	*n*	*M*_diff_	*t* (*df*)	*p*	*d*	*n*	*M*_diff_	*t* (*df*)	*p*	*d*
1–4	67	3.54	5.42 (66)	<.001	.68	106	2.14	3.76 (105)	<.001	.40	107	10.50	6.23 (106)	<.001	.50
5–8	92	2.38	4.18 (91)	<.001	.42	139	2.19	4.76 (138)	<.001	.34	138	6.49	4.95 (137)	<.001	.36
9–11	62	4.19	5.43 (61)	<.001	.64	99	2.49	5.47 (98)	<.001	.47	99	7.88	4.88 (98)	<.001	.39

268 Acorn mothers (75.9%) indicated they had learned a lot about parenting from attending Acorn, with 103 (76.3%) reaffirming this in the follow-up measure. Nearly all Acorn mothers (99.3%) indicated improvement in *Using a range of ways to interact with my child (e.g., through songs, games, activities to do together)* because of attending Acorn with 281 (79.6%) indicating “a great deal” or “quite a lot” of improvement. This was more pronounced for the second older attending child with 89.5% (17 mothers) indicating “a great deal” or “quite a lot” of improvement in this area. Over 9 in 10 Acorn mothers indicated they had improved across all of the remaining items addressing Objective 2 as a result of attending the program, with this being sustained 6–8 months later (Table 5).

Taking the 108 Acorn mothers who completed a post-measure and had older children (of any age) who had not attended Acorn, when asked *has Acorn helped with your parenting for any other children who did not attend*, 95 (87.9%) indicated in the affirmative with 40 (37.0%) indicating “a great deal”. Of the 47 mothers with older non-attending children who completed the follow-up interview, 33 (70.2%) indicated the program had helped in this area with 14 (29.8%) indicating “a great deal”.

Over 9 in 10 Acorn mothers reported improvements in both their *Ability to reflect on how my past experiences impact on my parenting* (with 242, indicating this improved “a great deal” or “quite a lot” because of attending Acorn), and their *Ability to reflect on how my current health impacts on my relationship with my child* (with 239, 67.7% indicating “a great deal” or “quite a lot” of improvement). These positive reported outcomes were consistently indicated six-eight months after completing the program (Table 5).

#### 5.3.3. Objective 3: to enhance mothers' wellbeing, coping skills, resilience, and self-efficacy

There are strong indications that the Acorn approach has provided holistic wellbeing benefits for engaged mothers. The PHQ-9 has demonstrated significant improvement in depression scores pre and post Acorn with a small-medium effect size (*M*_diff _= 2.27, *t*(343) = 8.90, *p* < .0001, *d* .37). All of the vulnerable subgroups considered improved their PHQ-9 scores significantly with small to medium effect sizes (Table 8). While mothers with BPD scored highest in both pre and post measures of depression, this reduced significantly with the largest effect size *d*.43. Improvements were significant at different time points throughout the program (Table 7). Clinically significant improvement (from a “clinical” to “non-clinical score”) were calculated for the 190 mothers scoring in the clinical range in the PHQ-9 pre-measure for whom post-measures were obtained; 50 of these mothers (26.3%) met these criteria for “clinically significant” improvement.

**Table 8:** Depression outcomes for acorn mothers and vulnerable subgroups.

PHQ-9	*N*	*n*	Pre		Post		*t* (*df*)	*p*	*d*
			*M*	*SD*	*M*	*SD*			
All	388	344	11.24	5.87	8.98	5.57	7.90 (343)	<.001	.38
Non-English spoken at Home	57	55	10.76	6.21	9.13	6.13	2.24 (54)	.029	.26
2+ children ≤36 months	52	47	11.26	5.90	8.91	5.51	3.34 (46)	<.005	.40
Single	59	53	11.47	7.00	9.09	6.02	3.18 (52)	.002	.34
Main household income Government Benefit	129	110	11.75	6.35	9.62	5.95	4.15 (109)	<.001	.34
School Level ≤ Year 12	100	87	11.48	5.95	9.40	5.53	3.58 (86)	.001	.35
BPD Indicated	89	79	14.16	6.48	11.39	5.27	4.14 (78)	<.001	.43

Taking those mothers who provided both pre- and post-measures, the number of Acorn mothers with “Minimal” or “No” depressive symptoms improved to a majority in the post-measure from 154 (44.8%) to 212 (61.7%); the number of mothers with no symptoms doubled to just over one in four. Reductions in “Major Depression moderately severe to severe” were also evident, from 97 (28.2%) indicating this in the pre-measure to 60 mothers (17.4%) in the post-measure. Those finding coping with their depressive symptoms “very” or “extremely” difficult also reduced by more than half, from 101 (29.4%) to 43 (12.5%).

There has also been a clear significant improvement for Acorn mothers in “Total Parenting Stress” and in each of the PSI-SF domains (Table 9). Taking those for whom pre and post measures were obtained, the numbers indicating “clinically significant” or “high” for “Total Parenting Stress” halved from 60 (17.4%) to 30 (8.7%), and for “Parental Distress” (the most prevalent stress related issue for participating mothers), the numbers in these levels moved from 190 (55.2%) to 144 (41.9%). These significant improvements with effect sizes ranging from medium to large were evident for a range of “most vulnerable” subgroups of Acorn mothers including those whose household income was mainly Government benefit and those with lower educational levels. Sixteen Acorn mothers scored in the 96th percentile or higher in the pre-measure of the “Parent-Child Dysfunctional Interaction” scale with this reducing to 4 in the post-measure.

**Table 9:** Parental stress outcomes for acorn mothers and vulnerable subgroups.

** **	*N*	*n*	Pre		Post		*t* (*df*)	*p*	*d*	*BCa 95% CI*
			*M*	*SD*	*M*	*SD*				
**PSI-SF: Total parenting stress**										
All	388	344	90.89	19.53	82.75	18.93	9.27 (343)	<.001	.42	—
Non-English spoken at Home	57	55	88.96	20.84	80.29	19.79	3.47 (54)	.001	.42	—
2+ children ≤36 months	52	45	96.16	18.86	84.24	16.27	4.34 (44)	<.001	.63	—
Single	59	53	90.51	23.30	82.08	19.48	3.77 (52)	<.001	.36	—
Main income Government Benefit	129	110	89.75	21.97	82.05	20.22	5.08 (109)	<.001	.35	—
School Level ≤ Year 12	100	86	89.92	19.98	80.83	18.32	5.15 (85)	<.001	.45	—
Second older attending Child*	22	16	100.06	19.90	81.93	24.85	3.52 (15)	.003	.91	*9.25, 26.52*
BPD Indicated	89	80	99.98	19.97	92.54	19.30	4.13 (79)	<.001	.37	—
**PSI-SF: Parental distress**										
All	388	344	38.44	8.49	35.09	8.22	8.58 (343)	<.001	.40	—
Non-English spoken at Home	57	55	37.45	9.07	33.73	10.0	3.31 (54)	.002	.41	—
2+ children ≤36 months	52	45	39.02	8.10	34.11	6.97	4.97 (44)	<.001	.61	—
Single	59	53	38.58	10.11	35.30	8.34	3.29 (52)	.002	.32	—
Main income Government Benefit	129	110	38.06	9.10	34.55	8.49	4.90 (109)	<.001	.39	—
School Level ≤ Year 12	100	86	38.17	8.53	34.64	7.66	4.53 (85)	<.001	.41	—
Second older attending Child*	22	16	38.69	9.25	33.56	10.03	3.50 (15)	.003	.55	*2.62, 7.63*
BPD Indicated	89	80	43.64	7.42	39.29	7.76	5.59 (79)	<.001	.59	—
**PSI-SF: Parent child dysfunctional interaction**										
All	388	344	24.54	7.72	21.76	6.96	7.71 (343)	<.001	.36	—
Non-English spoken at Home	57	55	24.10	7.20	20.98	6.87	3.07 (54)	.003	.42	—
2+ children ≤36 months	52	45	27.00	7.32	22.53	5.57	4.39 (44)	<.001	.61	—
Single	59	53	25.46	9.43	21.90	7.34	3.65 (52)	.001	.38	—
Main income Government Benefit	129	110	24.29	8.70	21.73	7.31	4.23 (109)	<.001	.29	—
School Level ≤ Year 12	100	86	24.28	8.23	20.99	7.19	4.24 (85)	<.001	.40	—
Second older attending Child*	22	16	28.43	7.71	22.19	8.93	3.74 (15)	.002	.81	*2.75, 8.06*
BPD Indicated	89	80	27.33	8.95	24.53	7.20	3.34 (79)	.001	.31	—
**PSI-SF: Difficult child**										
All	388	344	27.83	8.14	26.03	8.02	4.86 (343)	<.001	.22	—
Non-English spoken at Home	57	55	27.55	8.44	25.60	7.98	2.32 (54)	.024	.23	—
2+ children ≤36 months	52	45	30.58	9.28	27.67	8.81	1.98 (44)	.054	.31	—
Single	59	53	26.62	7.71	25.06	7.99	1.71 (52)	.093	.20	—
Main income Government Benefit	129	110	27.31	8.10	25.87	8.61	2.30 (109)	.019	.17	—
School Level ≤ Year 12	100	86	27.22	7.54	25.31	7.87	2.96 (85)	.004	.29	—
Second older attending Child*	22	16	32.94	9.14	26.19	10.97	2.77 (15)	.014	.62	*2.88, 10.82*
BPD Indicated	89	80	29.24	8.64	28.76	9.11	.66 (79)	.510	.05	—

* Bootstrapping applied.

Mothers who brought two children to the program appear to have benefitted most across the domains addressed by the PSI-SF with higher effect sizes evident in relation to both their younger and older attending children. “Total Parenting Stress” was noticeably higher in relation to the second older attending child on commencing Acorn but this improved substantially. Applying the bootstrapping technique, there was on average a decrease in this score from pre (*M* = 100.06, *SE* = 4.98) to post (*M* = 81.93, *SE* = 6.21), and this difference of 18.13, BCa 95% CI [9.25, 26.52] was significant *t*(15) = 3.52, *p* = .003, representing a large effect size *d* = .91.

Nearly nine in ten mothers indicated that the program *has helped me to cope better as a parent,* with 119 (33.8%) indicating “strongly agree” with this item. This included mothers indicating BPD with 72 (88.9%) agreeing that Acorn had helped them in this regard (30.9% “strongly” agreeing). The proportions of completing mothers “strongly” agreeing with this item improved as the program matured from 23.4% in Waves 1–4 to 40.4% in Waves 9–11. 319 (90.4%) mothers indicated that *the group has made me feel better about myself as a parent* (38.8% “strongly”) including 71 mothers indicating BPD (87.7%) with 29 (35.8%) of these mothers “strongly” agreeing.

While 233 (66.0%) endorsed the item, Acorn mothers were less likely to agree that *the group has made me more confident to access other family services if I need them*, with this lower level of agreement being partly explained by some mothers expressing existing confidence in this area prior to Acorn; this may be related to the fact that all mothers were referred to Acorn by existing services with which they had some degree of engagement. However, enhanced confidence to access suitable family services appeared to improve after completing Acorn and was accompanied by the almost universal recognition among Acorn mothers that *I am not alone in the struggles I can face as a parent*; 293 (83.0%) mothers indicated this had improved “a great deal” or “quite a lot” by the end of the program and 77.3% indicated this level of improvement six-eight months later.

While around one third of mothers did not indicate a desire to improve family independence and resilience prior to beginning the program, most mothers in the post-measure (208, 59.1%) indicated that the group had helped in this area and this appears to have improved months after completing the program (67.4%). There were sixty mothers indicating BPD who expressed the wish for their family to be more independent and resilient at the beginning of Acorn and thirty-eight of these (63.3%) agreed that the group had helped them in this regard in the post-measure.

#### 5.3.4. Objective 4: to expand and strengthen social/community supports and build social connectedness

Over eight in ten (295 mothers) agreed *I made friends with other parents in the group*, with 134 (38.1%) strongly agreeing this was the case. Strength of agreement improved as the program matured (from 33.9% “strongly” agreeing in Waves 1–4 to 44.4% in Waves 9–11) and was sustained 6–8 months later (Table 5). For mothers indicating BPD, 77.8% agreed they had made friends, and 39.5% strongly agreed this had occurred. Of the 53 single mothers who completed Acorn, 79.2% (42 mothers) agreed they had made friends at the end of the program, 34.0% “strongly”. Of the 56 completing mothers who spoke a language other than English at home, 78.6% (44 mothers) agreed they had made friends at the end of the program, 35.7% “strongly”.

Acorn has been more successful in fostering relationships and connections between attending mothers than in enhancing feelings of connectedness to their communities. However, 224 mothers, (63.6%) indicated *I feel more connected to my community because of attending the group*, with this proportion being similar for mothers indicating BPD (66.7%). Most mothers continued to endorse this change and attribute this to Acorn six-eight months after the program (Table 5).

It was also the case that 314 mothers (89.7%) indicated they “will share” their learnings from the Acorn program with their peers, and confirmation that this intention was enacted was indicated in the follow-up measure with 121 (89.6%) agreeing *I have shared what I have learnt from the group program with other parents like me*, (with just under half of these strongly agreeing this item). The intention to share learnings from Acorn were also signified by 82.1% of mothers from households that spoke a non-English language at home (41.1% “strongly” agreeing), and by single mothers (90.6%, and 30.2% respectively). Confirmation from these groups that this had occurred 6–8 months later was provided by 81.1% of non-English speaking household mothers and 77.8% of single mothers.

Most Acorn mothers (220, 62.3%) indicated that participation in the program *has helped our family to have better relationships, and* this appears to have improved over time; taking those 122 mothers for whom both post, and follow-up measures are available, 58.2% of these mothers indicated Acorn had helped to improved family relationships in the post measure with this increasing to 76.9% in the follow-up measure.

### 5.4. Program effects and attendance

The extent to which program attendance effected outcomes was explored by considering the 21 mothers who completed Acorn and provided pre and post Client self-completion questionnaires but attended 7 sessions or less (just under half the program). The profile of these mothers was very similar demographically to the larger group of mothers. Applying paired sample t tests for all psychometric scales where pre and post scores were available for these mothers, significant improvements were found for all the PSI-SF measures: “Total Parenting Stress” (*M*_diff _= 15.37, *t*(18) = 3.50, *p* < .005, *d* .76); “Parental Distress” (*M*_diff _= 6.00, *t*(18) = 4.00, *p* < .001, *d* .63); “Parent-Child Dysfunctional Interaction” (*M*_diff _= 3.16, *t*(18) = 2.51, *p* < .05, *d* .45); “Difficult Child” (*M*_diff _= 4.58, *t*(18) = 2.17, *p* < .05, *d* .49). However, improvement in depression scores was not significant for these mothers, and for the 16 mothers whose children were aged 1 year or less Perceived Parental Self-Efficacy (KPCS) did not improve significantly. Only two of the mothers who had attended 7 times or less completed pre NCAT observations. Mothers who completed the program but attended less than half of the Acorn sessions reported improvements across all the indicators addressing each objective listed in Table 5 which largely mirrored the answers supplied by the broader group.

## 6. Discussion

It has been documented that group-based interventions addressing maternal mental health can record poor attendances with high drop-out rates (69). However, despite the requirement to commute to the program at set times each week with their child(ren), Acorn has successfully encouraged engagement for the majority of even the most vulnerable of its targeted population many of whom living with profound comorbid mental illness. The program is meeting the needs of its client base with its objectives clearly aligned with mothers' personal objectives for attending, and Acorn mothers broadly acknowledged and appreciated the helpfulness and delivery of the program's three principal components. From the Acorn mothers' perspective, the program has provided an environment that felt safe, appropriate, and respectful, with clear resources and explanations, using facilitators who were almost universally perceived as understanding of client issues, and presenting opportunities for group-based peer support. Many of these program delivery elements harmonise with previously identified service provision wishes of mothers experiencing postpartum mental illness (147). Subsequently, Acorn mothers almost universally indicated they would recommend the program to their peers after completing Acorn, and most indicated they had shared learnings with peers six-eight months later. The importance of client preference in choosing appropriate mental health therapies in order to optimise their engagement has been highlighted (148), and the willingness of Acorn mothers to discuss their experiences of this valued program with peers may have broader benefits in terms of extending its reach to those who may otherwise be more difficult to engage, including those from linguistically diverse backgrounds (149).

The program has blended evidence-based strategies that have successfully enhanced the quality of the mother-child interaction. NCAT observations applied to mother, child, and their interaction, found significant improvement evident in all three measures and these were triangulated with mother's self-reports which indicated related beneficial and lasting improvement. It is also noteworthy that the NCAT “Caregiver Total” has strong predictive validity with measures of “maternal support”, and measures of “maternal reflective functioning” (63, 137, 140, 141), and the latter is consistent with Acorn mothers' self-reports of improved ability to reflect on the impacts of their past experiences and current mental health on their relationship with their child and their parenting. “Caregiver-Infant Total” scores have been found to predict security of attachment (150) and improvements in this measure of the quality of the dyad interaction were significant across a range of “most” vulnerable subgroups taken from what can be considered an already vulnerable target population of mothers and children. Meta-analysis has also established the NCAT as a reliable outcome measure for interventions addressing parent sensitivity (151); a central parenting concept in attachment theory and a key determinant to promote parent-child attachment (37, 152). There were significant improvements in the subscale “sensitivity to cues”, and while this should be interpreted with some caution, this is consistent with the three more reliable composite scales and self-reported improvements in identifying their child's needs and responding appropriately. While the above improvements are encouraging across NCAT measures, many of the dyads began the program at a low baseline level and around one quarter of the dyads remained >−1 SD below the normative mean in the “Caregiver-Infant Total” post-measure. However, it was noteworthy that improvements in the child interactive behaviours with their mother were substantial and resulted in similar proportions scoring >−1SD in the “Child Total” post-observations to that of the normative population. This was largely influenced by significant improvements in the child's “clarity of cues” during the interaction which may be attributed to their engagement in Acorn dance play activities.

However, improvements in the quality of the dyad interaction were not found to be significant for those who spoke a language other than English at home. Interpretation of why the “non-English speaking household” dyads did not improve significantly is problematic; this finding contrasts with the significant improvements found for this subgroup in PPSE, and their self-reported improvements in parenting interaction. For example, 89.3% of mothers from non-English speaking homes agreed that they interacted better with their child because of Acorn in the post measure (39.3% “strongly”), and 100% that the group had helped them to feel closer to their child (50% “strongly”). However, this small NCAT group of 19 observed dyads were culturally heterogeneous (speaking 7 different languages at home), and while the NCAT has been successfully applied with some ethnic communities it is possible that cultural barriers to be being observed and videoed with their children may have influenced behaviour for some of these dyads.

The large majority of mothers indicated that they interacted better with their children because of the program, that their child had benefitted, and that the program had helped them feel closer to their child, and these positive indications endured six-eight months after completion. These benefits were also evident regarding the second older attending child to the program, and mothers almost universally indicated that the program has helped in their parenting of other non-attending children. The majority of Acorn mothers reported improving substantially across a range of parenting qualities including understanding their child's needs, feelings, and perspectives of the world and enhancing their ability to respond to their child appropriately, with these being reaffirmed six-eight months after leaving Acorn. Given the difficulties maternal mental illness can impose on mother-child interaction the above self-reported improvements are notable. These were accompanied by widespread reporting of improved (and sustained) parental confidence along with significant improvements in perceived parental self-efficacy (PPSE) with medium effect sizes for Acorn mothers across all the vulnerable subgroupings considered, including those for whom Government benefits were the main source of household income and those with low formal education (known moderators of the effects of maternal mental illness on parenting behaviour). Given the importance of PPSE for successful parenting and its relationship to actual parenting competence this is very encouraging and supports the substantial reported improvements in parental competence indicated by over 9 in 10 Acorn mothers both on completing the program and six-eight months later. These included employing a range of new ways to interact with their child, and feeling more comfortable with interactive playing and child exploration.

The standardized tools applied in this evaluation provide clear evidence that the well-being of most mothers attending Acorn has improved in the dimensions addressed. Additional self-reported indications provided after program completion and six-eight months later corroborate this improvement with mothers attributing this to their participation in the program. Many reported that they felt better about themselves as parents and were better able to cope as a parent (with substantial numbers also indicating that they were more able to cope with their depressive symptoms on the PHQ-9). While many participating mothers experienced clinically significant improvements in depression and PPSE, we support the view that in promoting the wellbeing of mothers with mental illnesses, reducing sub-clinical symptoms for mothers with mental health difficulties is also important (153) and this was broadly evident. In reference to the mental health movement conceptualisation of mental health “recovery” as a personal process above and beyond symptom relief, and involving the restoration of functioning (154), the burden of evidence here attests to the value of the Acorn program. Moreover, given the known protective buffering effects of improved parent-child relationships and parental confidence, it is likely that for those mothers experiencing on-going maternal mental health issues, there are still benefits acquired from the program in terms of well-being improvement and reducing possible detrimental impacts of their mental health problems on their children.

There were also significant improvements in “Total Parental Stress”, and in the “Parental Distress”, “Parent-Child Dysfunctional Interaction” and “Difficult Child” dimensions addressed by the PSI-SF, with those mothers who brought a second older eligible child to the program appearing to acquire greater benefit from the program in relation to this older child. Improvement in these dimensions of parental stress were identified across the vulnerable subgroupings considered. However, for the “Difficult Child” subscale, single mothers and those with two children aged 36 months or less did not reach statistically significant levels of improvement for their youngest child, although this was marginal (*p* = .093 and *p* = .054 respectively). In the latter case, there were 29 completing “Acorn mothers” with an older eligible child who did not attend Acorn. Given that the largest effect size for “Difficult Child” improvement was in relation to mothers with a second older attending child, further measures to encourage mothers to bring all their eligible children to the program could prove beneficial in this dimension. “Difficult Child” addresses the mother's perception of the child's temperament, defiance, noncompliance and demandingness, but this can be influenced by “external” factors (155), and it is possible that latent influences on the perceptions of some single mothers has dampened program impacts for this measure. More notably however, there was no significant improvement in the “Difficult Child” measure for mothers indicting BPD following participation in Acorn. Mothers with BPD have been found to be more likely to perceive their parental stress as being related to their child (156), and as many of the Acorn mothers indicting BPD were not previously diagnosed, it is possible that they had low knowledge of their illness and little insight into its possible influence on these perceptions making improvement in this area for these mothers more challenging. However, 80.0% of those Acorn mothers indicating BPD, scored within “normal” limits in the post-measure for the “Difficult Child” scale. It is also noteworthy that 12 of the 16 Acorn mothers who scored above the 96th percentile in the “Parent-Child Dysfunctional Interaction” scale pre-measure improved their score to move away from this indication of potential child abuse in the post-measure (130). This unexpected outcome highlights the importance of significant improvements evident in this dimension for Acorn mothers as a whole and for all of the vulnerable subgroups considered.

Improvements in family relationships, independence and resilience were also noted by most mothers with indications that these continued to improve months after the program was completed. This suggests that for some mothers, these improvements may have taken time to come to fruition after the program, but that where this was the case, these mothers nonetheless still identified and acknowledged the role of Acorn in helping this to happen. There is evidence that mental health programs that include partners benefit families (46) and future program adaptations in this regard could be considered. However, taken with the indications that Acorn had helped mothers in their parenting of non-attending children, participation in the program already appears to have precipitated improvements in family life for substantial numbers of engaged women.

Social connectivity has been enhanced and sustained for Acorn mothers including for those subgroups who may experience greater levels of social isolation, and improved recognition that participants were not alone in their parental struggles was broadly indicated following Acorn. This finding addresses an identified need for interventions that nurture social connectedness for migrant mothers of young children revealed in recent qualitative work (157). Assembling mothers with shared parenting and mental health issues and engaging them in collective interactive group activities has encouraged friendships to develop for over 8 in 10 participants including single mothers, mothers indicating BPD, and those speaking a language other than English at home. This has met a personal objective for attending Acorn for most participants and most considered being able to talk to other mothers in the group to be a “huge help”. Many of these friendships have continued; similar proportions of mothers indicated this in the follow-up interviews and around 4 in 10 “strongly” agreed this was the case. These outcomes are pertinent given evidence that improving social connectedness benefits mental health generally (158, 159) and depression specifically (160–163). There is also qualitative evidence that access to peer support promotes emotional wellbeing for mothers experiencing perinatal mental illness by helping to alleviate isolation, guilt and stigma (165). Given that there are known associations of social isolation and disconnect with maternal mental ill health, the program has clearly provided benefits in this regard.

Finally, Acorn mothers attending less than half of the Acorn sessions appear to still benefit from the program, notably in reducing dimensions of parental stress, and across the range of self-reported measures but not in significantly in PPSE or their depression measure. While small numbers render interpretations tentative, clearly mothers who for whatever reason cannot attend the whole program are still experiencing positive outcomes. While these mothers attended less sessions, their attendance was however spread over the duration of the program, allowing time to enact strategies at home at their own pace between attended sessions. It is also the case that providing the current program over the three months may be particularly important as most Acorn mothers were unable to attend all 15 sessions.

## 7. Study limitations and strengths

The identification of an appropriate instrument to observe the quality of the parent-child interaction was problematic, and we were particularly concerned to avoid a potential disincentive to participation for targeted mothers known to be difficult to engage. Once selected, the need for training and accreditation for Mental Health Clinicians to apply the NCAT in the homes of participants further delayed its application and its adoption also required substantial additional resources and time. Following a change of management and staffing at the host agency, the NCAT was not applied in Wave 11. However, the demographic and psychological profile of the 145 dyads completing these observations was very similar to that of all attending mothers, and this tended to be consistent across all Acorn waves (a consequence of maintaining consistent referral pathways over the duration of the program).

We were mindful of overburdening or alienating vulnerable women in the community setting with the demands of evaluation and focused specifically on tools that addressed the objectives of Acorn. The program did not screen for substance use disorder, and while the clinical diagnosis of psychotic disorder was an exclusion criterion, the program relied on referrers to identify this condition. We did not screen for bipolar disorder, the presence of which may interfere with the score for the MSI-BPD (136). We did not measure post-traumatic stress disorder or anxiety, and while we asked Acorn mothers about resilience in their family, we did not apply a quantitative measure of client resilience which we explored qualitatively as part of a supplemental study (165). We decided to screen for BPD in Wave 4 following revelations in the Critical Reference Group that some referred mothers leaving hospital had been diagnosed with this condition. It has been estimated that up to 23% of those who use outpatient psychiatric services have BPD (166) and given our findings that 30.4% of enrolled mothers indicated BPD, the prevalence of this condition among mothers with maternal mental ill health may be higher. There are of course a range of alternative tools and dimensions which could have been included to measure “wellbeing” and these could be usefully explored in the future.

Comparisons between post-measures and follow-up interviews employed different methods to obtain responses from Acorn mothers. It is possible that respondents may have been more inclined to provide positive answers in the follow-up because of these being elicited in an interview as opposed to the demonstrable anonymity of a self-completion questionnaire. However, given that the interviewer for the follow-up was independent, and that all mothers were clearly informed their responses and names would remain strictly confidential, participants had little motivation to provide misinformation about positive outcomes in their personal lives attributed to participating in a program they had completed months earlier. Moreover, it may have been expected that the perceived impact of a program would wane over time or possibly a contrast effect might occur whereby current impacts would be judged inferior in comparison to the largely positive impressions experienced immediately after the program. However, arguably the most striking finding has been the consistency of responses between the two measures taken six to eight months apart, and we are confident that this consistency coupled with the above considerations have yielded authentic responses from mothers.

We employed an action research design for this evaluation which precluded using a comparison/control group. Given the heterogeneity of the client base, the range of impacts sought reflected in the objectives, and the combination of different program strategies to achieve them, the importance of utilizing the evaluation formatively was recognised in anticipation that program processes and outcomes would need to be monitored closely to inform potential program revision. There were ethical concerns raised by stakeholders about withholding the intervention from some vulnerable mothers and their young children for the purpose of establishing a comparison group. There was no “waiting list” available as the program expanded to accommodate all referrals. Causal attribution was premised on triangulating mixed methods addressing a range of dimensions germane to parental wellbeing and the quality of the parenting relationship articulated in Acorn's program logic. Participant own judgements of causality were obtained through the Client Self-Completion Questionnaires and through follow-up interviews in which former clients could personally reflect on how the program had (or had not) achieved its objectives in relation to their own lives. A range of deeply personal positive improvements were broadly attributed to the program, and often strongly indicated. Given the profound challenges experienced by Acorn mothers in the most intimate of their human relationships, providing this evidence supports the effectiveness of the Acorn program. While future studies may be able to include a waiting list control or randomised control design to establish efficacy, the case that the Acorn program has achieved its stated goal and objectives is strongly supported by this evaluation.

There are several strengths to this study. The action research design of the evaluation enabled on-going monitoring, timely feedback, reflection and refinements to be integrated into this community program over a long period of time between “Waves” of delivery. The evaluation design also enabled the service providers to be enlisted in the efficient collection of data across the geographically dispersed groups of this multi-site program. While the three principal components remained constant throughout, supplemental adjustments to the program, its delivery and its evaluation were flexibly facilitated resulting in a matured program model detailed in its completed Manual (116). Participant indications of process improvements resulting from this endeavour over the duration of the program are evident, and particularly improved client engagement and retention as the program matured. This was accompanied by consistently highly significant improvements across the three applied standardized self-completion measures throughout the duration of the program (Table 7). It is notable that the effect size for each of the these measures tended to be lower around the middle of the program during Waves 5–8, a period when three Mental Health Clinicians left the program and were replaced with new workers who required a period of induction. However, the strength of self-reported positive endorsement clearly improved in terms of perceived child benefits, improved parent-child interaction, improved parental confidence and coping, and establishing friendships. We therefore conclude that these process and outcome improvements are related and were nurtured by the formative use of action research in the evaluation.

The application of mixed methods in this study has provided comprehensive, consistent and triangulated evidence of the suitability of the program and its array of lasting beneficial impacts. The inclusion of direct standardized observations of the quality of the parenting interaction also answers the call to implement measures of parenting quality which are not wholly reliant on mothers' self reports (85) and for studies that treat depressed mothers to include measures that address benefits for children (76). A major strength of this study is the exploration of a range of program impacts for a heterogeneous community with sub-analyses addressing known at risk groups.

## 8. Conclusion

The evaluation findings lend support to growing evidence of the benefits of psychotherapy approaches to enhancing mental health wellbeing in community settings (69, 167–169). Moreover, this evaluation demonstrates that by integrating specific evidence-based strategies within a holistic group-based community program that addresses both maternal mental health wellbeing and the mother-infant relationship, substantial benefits to both can be acquired for a diverse population of mothers and a range of specific “at risk” groups of vulnerable mothers experiencing mental health issues, their children and families. To our knowledge this is the first study that examines the effectiveness of a single program for the range of subgroups of mothers with maternal mental health issues we have identified. Many mothers in this study had mental health difficulties which could be considered to be at the severe end of the spectrum, and while some dyads may require further support, the Acorn program has succeeded in its goal of holistically nurturing and enhancing the wellbeing of those most vulnerable of mothers experiencing maternal mental illness and promoting a sustained quality of their relationship with their children. All Acorn mothers were referred by existing mental health services, but the program has enhanced and added value to their mental wellbeing, parenting and parenting enjoyment including for mothers from ethnically diverse communities who often experience additional barriers to engagement with maternal mental health services (170).

Given the global prevalence of maternal mental ill health and the alarming expansion in numbers of those experiencing greater parental stress, isolation and mental illness in the shadow of COVID-19 (171, 172), the increasing numbers of previously undiagnosed cases of maternal depression emerging through the implementation and expansion of routine screening procedures (173, 174), and the profound inter-generational consequences of poor quality parenting, this refined, highly appreciated and effective Acorn program model offers the potential to strengthen the mental health system's response for a diversity of families. However, it is still the case that many families living in rural and remote areas may be restricted from participating in a program that congregates mothers and children at the same geographical venue. These mothers may be at elevated risk of maternal mental illness (106, 175). As a future development, alternative “distance” modes of delivery for Acorn could be developed and evaluated to assess the engagement and effectiveness of this program for these relatively isolated families.

## Data Availability

The datasets presented in this article are not readily available because of Ethics Committee requirements. Requests to access the datasets should be directed to paul.aylward@torrens.edu.au.
